# Case of Septic Endocarditis with Cerebral Embolism

**Published:** 1891-06

**Authors:** F. H. Edgeworth

**Affiliations:** Physician to the Bristol Hospital for Sick Children and Women


### CASE OF SEPTIC ENDOCARDITIS WITH CEREBRAL EMBOLISM.

BY

F. H. Edgeworth, M.B., B.C., B.A. Cantab., B.Sc. Lond.,

*Physician to the Bristol Hospital for Sick Children and Women.*

---

Bessie E., aged ten years, was admitted to the Children's Hospital on March 25th, 1890, under the care of Dr. Roué, whom I thank for permission to publish the case.

For five or six weeks previous to admission the patient had suffered from pain in both shoulders, knees, and ankles—severe enough for her to keep to the sofa.

On admission, patient was free from pain in the joints; and no joint effusion or tenderness could be detected. On examining the chest, the apex-beat was felt in the fourth left space, just outside the nipple-line; and on auscultation, a mitral systolic bruit was heard. The lungs, abdomen, and urine, were normal. The temperature was 99.2°.

The diagnosis was rheumatic arthritis and endocarditis.

The history of the case up to July 14th can be quickly passed over. The patient, in spite of all treatment, had recurring attacks of subacute arthritis of various joints, and became, as time went on, more and more anæmic. The only important point to notice is, that on April 19th there was suddenly pain in the splenic region; but on palpation, no enlargement or tenderness of the spleen could be made out.

At the beginning of July, on Dr. Roué going away, the patient came under my care.

On July 10th a double pericardial friction-sound was heard over the innermost inch of the second left intercostal space: this persisted, but no physical signs of pericardial effusion ever developed.

In the evening of July 14th the patient suddenly screamed out and became unconscious. No convulsive movements were seen. On the next day the patient was partially, and on the following completely, conscious. On examination, it was found that the condition was as follows:—Eyes normal in direction, and can be freely moved. Complete right motor hemiplegia—face, tongue, arm, and leg. Patient cannot speak. There is no anæsthesia of the right side. Patient understands what is said, and can see quite well with both eyes. The skin reflexes are normal and equal on both sides of the body: on the right side the tendon-jerks are increased, and there is ankle- but no rectus-clonus. In the fundus of the left eye two hemorrhages are seen—one, to the inner and lower side of the disk, round and dark-red; the other, larger, to the outer side of the disk, round with a white centre: the edges of the disk are not clear. In the fundus of the right eye there are no hemorrhages: the edges of the disk are not clear. These appearances, it may be said, persisted up to the time of death. No definite optic neuritis developed.

The temperature, which up to July 14th had generally been normal in the morning, but going up to 101° and occasionally to 102° in the evening, went up to 101.8° at the time of the attack, and never became normal again, generally varying from 100°–101°.

The discovery of this retinal hemorrhage with a white centre led to the opinion that septic embolism of a retinal artery had occurred, and this to the supposition that the rheumatic had passed on into a septic endocarditis with subsequent septic embolism of the left middle cerebral artery. As to the seat of embolism in the brain, two suppositions were open—embolism either of the first three cortical branches of the left middle cerebral, or of its central branches leading to lesion of the anterior two-thirds of the posterior limb of the internal capsule.

The further progress of the case may be very shortly described. The right hemiplegia persisted, and in a couple of weeks slight rigidity of the right arm was added. The motor aphasia also persisted: the child never said more than "No" and "Very thirsty" up to the time of her death. At the beginning of August the patient began to move the right leg a little. On August 10th she suddenly became unconscious, with clonic convulsions of the left leg and right arm and leg, lasting a few minutes, and followed by rigidity in all the limbs. Three similar attacks of clonic convulsions recurred, and the child died in a couple of hours without regaining consciousness.

On making a *post mortem* examination on the next day, I found the following interesting appearances:—In the abdomen, the intestines were healthy, as were also the liver and both kidneys. In the spleen was a small old infarct. In the thorax, the lungs were normal: there were ʒii. of clear yellow fluid in the pericardium, with a very slight deposit of lymph on the anterior surface of the right auricle and opposite surface of visceral layer of pericardium (this corresponded in position to the friction-sound heard during life); elsewhere the pericardium was normal. The right side of the heart was normal: on the left side, the left ventricle was somewhat dilated; the aortic valves were normal. On the mitral valves, which were incompetent, were large vegetations, but with no positive sign of ulcerative processes. On the posterior surface of the dilated left auricle was a patch, as large as a shilling, of ulcerative endocarditis. (The heart, it may be mentioned, is in the museum of the Children's Hospital.)

On removing the brain, there was seen a purple blood-clot under the pia mater over the inferior vermiform process of the cerebellum. The cerebral cortex was yellow in colour; scattered generally in the pia-arachnoid over the external surface of both hemispheres were spots of yellowish lymph, in size varying from a pin's head to that of a pea. The left Sylvian artery with its four cortical branches were normal, and contained no embolus; and there was no softening of any portion of the left hemisphere. On the right Sylvian artery was an aneurism as large as a small marble. The arteries beyond were normal, and contained blood; and there was no softening of the cortex supplied by them. On incising the brain, there was seen a large clot in the right hemisphere, extending upwards into the centrum ovale minus, downwards nearly to the apex of the temporo-sphenoidal lobe, destroying nearly the whole of the right caudate and lenticular nuclei and the internal capsule, and rupturing into the right lateral ventricle. No ruptured arteries could be seen. On the left side, there was a fœtid abscess with ragged walls occupying the position of the anterior limb and the front half of the posterior limb of the internal capsule, extending outwards into the nucleus lenticularis. The cavity was oval in shape, with its long axis measuring 1½ inches. Continuous with the blood-clot in the left side of the brain, was blood-clot filling up both lateral ventricles and the central canal as far down as the fourth ventricle, to the foramen of Majendie, through the roof of which it had passed, and spread on to the surface of the inferior vermiform process of the cerebellum, so giving rise to the appearance seen on examining the outside of the brain. This clot could be removed entire, forming a good cast of the lateral ventricles and central canal of the nervous system.

The rest of the brain was normal to naked-eye examination.

From the results of the *post mortem* examination, it seems clear that the case was, as supposed, at first one of rheumatic endocarditis,—during this time embolism of a splenic artery, with resulting infarct, occurred,—and that it subsequently became a septic endocarditis. Then came, suddenly, septic embolism of a left retinal artery (producing a retinal hemorrhage with a white centre) and of the lenticulo-striate branches of the left middle cerebral (producing a fœtid abscess).

When embolism of the right middle cerebral occurred is not certain; but that it did seems most probable, for the aneurism of the right Sylvian, and the rupture of the lenticulo-striate branches of the right middle cerebral were both probably the result of previous embolism.

The lesion of the right internal capsule was such, that its effects corresponded exactly to those of the first three cortical branches of the middle cerebral, so that during life there were no means of determining which was the site of the lesion.

